# Landscape of Molecular Crosstalk Perturbation between Lung Cancer and COVID-19

**DOI:** 10.3390/ijerph19063454

**Published:** 2022-03-15

**Authors:** Aditi Kuchi, Jiande Wu, Jyotsna Fuloria, Chindo Hicks

**Affiliations:** 1 Department of Genetics and the Bioinformatics and Genomics Program, Louisiana State University Health Sciences Center, School of Medicine, 533 Bolivar Street, New Orleans, LA 70112, USA; akuchi@lsuhsc.edu (A.K.); jwu2@lsuhsc.edu (J.W.); 2 University Medical Center New Orleans, 2000 Canal Street, New Orleans, LA 70112, USA; jyotsna.fuloria@lcmchealth.org

**Keywords:** coronavirus, COVID-19, SARS-CoV-2, gene expression, lung cancer, networks, signaling pathways

## Abstract

Background: Lung cancer patients have the worst outcomes when affected by coronavirus disease 2019 (COVID-19). The molecular mechanisms underlying the association between lung cancer and COVID-19 remain unknown. The objective of this investigation was to determine whether there is crosstalk in molecular perturbation between COVID-19 and lung cancer, and to identify a molecular signature, molecular networks and signaling pathways shared by the two diseases. Methods: We analyzed publicly available gene expression data from 52 severely affected COVID-19 human lung samples, 594 lung tumor samples and 54 normal disease-free lung samples. We performed network and pathways analysis to identify molecular networks and signaling pathways shared by the two diseases. Results: The investigation revealed a signature of genes associated with both diseases and signatures of genes uniquely associated with each disease, confirming crosstalk in molecular perturbation between COVID-19 and lung cancer. In addition, the analysis revealed molecular networks and signaling pathways associated with both diseases. Conclusions: The investigation revealed crosstalk in molecular perturbation between COVID-19 and lung cancer, and molecular networks and signaling pathways associated with the two diseases. Further research on a population impacted by both diseases is recommended to elucidate molecular drivers of the association between the two diseases.

## 1. Introduction

Coronavirus disease 2019 (COVID-19), caused by severe acute respiratory syndrome coronavirus 2 (SARS-CoV-2), is a worldwide pandemic that has caused unprecedented loss of human life and devastated the world economy [1,2,3]. Despite remarkable progress in the management of patients, the disease continues to cause devastation and overwhelming health care systems, as the increasing number of COVID-19-positive patients who require hospitalization and intensive care support continues to rise worldwide [1,2,3]. This dislocation of the global health care infrastructure is of particular concern in clinical management and treatment of patients with underlying chronic diseases, such as lung cancer [4,5]. Although currently there is no definitive data showing that COVID-19 causes lung cancer, emerging evidence from early studies has shown that lung cancer patients have almost twice the risk of SARS-CoV-2 infection compared to the general population [6,7]. However, it is not clear from the published reports whether there is crosstalk in molecular perturbation between COVID-19 and lung cancer. 

In a clinical epidemiology study conducted among 102 patients with lung cancer and COVID-19, researchers at the Memorial Sloan Kettering Cancer Center in New York examined the course of disease, impact of anti-tumor treatment and determinants of COVID-19 severity and recovery [6]. These investigators found that the severity of COVID-19 was high in lung cancer patients, with 62% of patients being hospitalized and 25% dead [6]. These results have been corroborated by results from a retrospective case study conducted across three hospitals in Wuhan China, which involved 28 cancer patients suffering from COVID-19 [7]. In that study, the investigators found that lung cancer was the most common cancer type in that group (25%) [7]. These observations must be balanced against the recognition that many lung cancer patients are older and have compromised immune systems resulting from underlying lung disease and decreased lung capacity, both of which are risk factors for COVID-19 [5]. Indeed, because of their suppressed immune systems resulting from chemotherapy, lung cancer patients may be at high risk of developing severe pulmonary complications due to COVID-19, which could lead to poorer clinical outcomes. In fact, accumulating evidence from clinical and epidemiological studies suggests that patients undergoing lung cancer treatment who are immunosuppressed from chemotherapy are more likely to be infected with SARS-CoV-2 and develop severe complications [6,7]. However, although our understanding of SARS-CoV-2 has undergone a huge leap since its outbreak, and vaccines have been developed at breakneck speed, the association between COVID-19 and lung cancer remains poorly understood. Understanding molecular crosstalk perturbation between COVID-19 and lung cancer has the promise to improve clinical management of lung cancer in the COVID-19 pandemic era. 

Epidemiological studies have shown that the clinical manifestation and severity of COVID-19 involves a broad range of symptoms, including fever, inflammation, cough and shortness of breath [1,2]. For example, patients with severe COVID-19 admitted to the intensive care unit are more likely to have proinflammatory cytokines, such as IFN-γ, IP-10, MCP-1, IL-1β, IL-4 IL-6 and IL-10, which drive the cytokine storm [8,9,10]. Severely ill patients may have poor disease course that progresses rapidly to multiple organ dysfunction and even death [11,12], and those who have shortness of breath can quickly progress into acute respiratory distress syndrome (ARDS) and suffer multiple organ dysfunction or even death within a short period from the time of diagnosis [13,14,15]. Interestingly, many of the symptoms exhibited by COVID-19 patients, such as cough, shortness of breath and inflammation, are also exhibited by lung cancer patients [6,7]. Lung cancer patients receiving immunotherapy with checkpoint blockade may have increased rates of complications from COVID-19-related inflammatory changes in the lung [7]. Moreover, the severity of COVID-19 in patients with lung cancer may be exacerbated by a comprised immune system. Taken together, these observations call for an urgent need to understand the crosstalk in molecular perturbation between COVID-19 and lung cancer to improve clinical management of lung cancer patients in the COVID-19 pandemic era. 

Since the outbreak of the pandemic, remarkable progress has been made in understanding the genomic basis of COVID-19 [16,17]. The SARS-CoV-2 genome has been sequenced [16], and the COVID-19 transcriptome has been mapped [17]. These advances have increased our understanding of the molecular taxonomy of SARS-CoV-2 and led to successful development of COVID-19 vaccines [18,19,20]. Yet, despite this remarkable progress, significant challenges remain. One of the more significant challenges is the lack of understanding of the molecular mechanisms associating COVID-19 with lung cancer and a discovery of clinically actionable biomarkers to guide clinical management of lung cancer patients in the COVID-19 pandemic era. The objective of this investigation was to characterize the landscape of molecular crosstalk perturbation between COVID-19 and lung cancer and to identify molecular networks and signaling pathways associating the two diseases. Our working hypothesis was that alterations in the transcriptome in COVID-19- and cancer-affected lungs could identify a signature of functionally related genes associating COVID-19 with lung cancer. We further hypothesized that COVID-19 and lung cancer have shared gene regulatory networks and signaling pathways, which potentially exacerbates the severity of COVID-19 in lung cancer patients. We addressed these hypotheses using publicly available gene expression data derived from lung tissue from patients severely affected with COVID-19 who succumbed to the disease, patients with lung tumors and disease-free control lung tissue.

## 2. Materials and Methods

**Figure 1:**
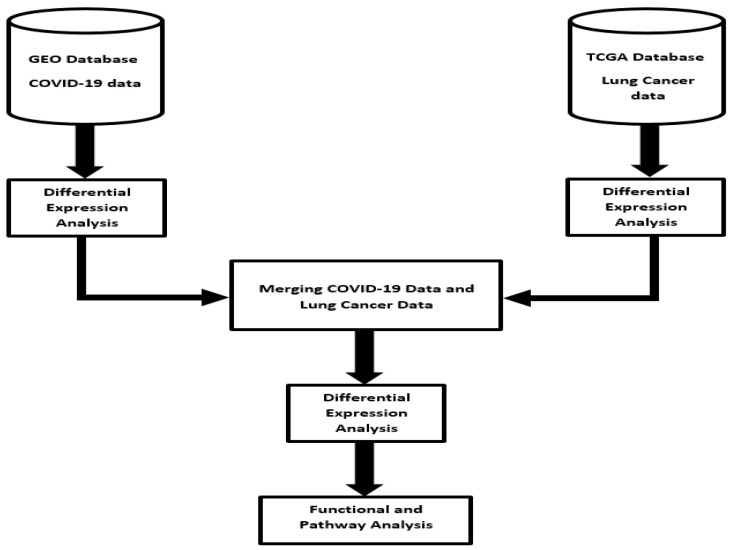
Project design and integrated data analysis workflow of gene expression data from COVID-19, lung cancer and normal lung samples. RNA-Seq data sets were downloaded from the Gene Expression Omnibus (GEO) and The Cancer Genome Atlas (TCGA).

Our experimental design approach focused on identifying molecular signatures of genes associated with both diseases, signatures of genes uniquely associated with each disease and molecular networks and signaling pathways associated with both diseases. The scientific premise and rationale was that among the genes transcriptionally associated with each disease, a subset of them are associated with both COVID-19 and lung cancer. Thus, molecular crosstalk perturbation between lung cancer and COVID-19 was considered an emergent property of functionally related genes transcriptionally associated with both diseases, interacting in gene regulatory networks and signaling pathways shared by the two diseases. We addressed this knowledge gap using an integrative genomic data analysis approach, combining RNA-Seq data derived from lung tissue of patients severely affected by COVID-19 who succumbed to the disease, lung tissue from patients affected by lung cancer and disease-free normal lung tissue controls. The overall project design and execution workflow, along with sources of RNA-Seq data, are presented in Figure 1. This section provides a brief but detailed description of sources of data and analysis strategies employed in this investigation.

### 2.1. Sources of Gene Expression Data on COVID-19 and Lung Cancer

Gene expression (RNA-Seq) data on COVID-19-affected lung samples (N = 52 samples) were derived from human lung autopsy tissue at the Massachusetts General Hospital and Columbia University Irving Medical Center in New York [21]. Processed data (sequence read counts) along with associated clinical information were downloaded from the Gene Expression Omnibus (GEO) database https://www.ncbi.nlm.nih.gov/geo/ (accessed on 10 February 2022) under accession # GSE150316 [21]. The data set was generated using the Illumina sequencing platform. Details about sample collection, processing, quality control and preparation for sequencing have been published elsewhere by the data originators [21]. Briefly, gene expression data was derived from lung tissue harvested from patients severely affected by COVID-19, who succumbed to the disease and underwent autopsy upon consent for clinical care [21]. All patients were confirmed for SARS-CoV-2 infection through qRT-PCR assays performed by the data originators [21].

Gene expression (RNA-Seq) data on 594 lung tumor samples and 52 control samples along with clinical information were downloaded from The Cancer Genome Atlas (TCGA) [22] via the Genomics Data Commons (GDC) https://portal.gdc.cancer.gov/ (accessed on 10 February 2022) using the data transfer tool [23]. The tumor samples were matched with clinical information for ascertainment of gene expression data. We processed the two data sets and checked them for quality. We then combined the two data sets to create one data matrix with three sample groups (COVID-19, lung tumors and normal lung samples). We performed noise reduction on the combined data set by filtering the genes with zero and very low expression values across samples. The resulting data set was normalized with quantile normalization using R Bioconductor implemented in our RNA-Sequence data analysis pipeline [24,25].

### 2.2. Bioinformatics Data Analysis

Using normalized data, we performed analysis comparing gene expression levels between COVID-19-affected and normal lung samples and between lung-cancer-affected and normal lung samples by computing *p*-values using the Limma package implemented in R [24,25]. This unbiased analysis was conducted to identify a signature of genes associated with COVID-19 and signature of genes associated with lung cancer. We then combined the two sets of genes to identify a signature of genes associated with both COVID-19 and lung cancer, and signatures of genes uniquely associated with each disease. The distribution of genes in the three gene signatures was organized using a Venn diagram. We performed additional analysis using gene expression data on genes associated with both diseases, by comparing their expression levels between COVID-19 lung and lung tumor samples to determine their direction of change, which was characterized as either up or downregulated. For each analysis, we controlled for multiple hypothesis testing using the false discovery rate (FDR) procedure [26]. In addition to estimates of *p*-values, we computed the log2 Fold Change (Log2 FC), defined as the median of gene expression values minus the gene expression value for each gene. The logFC was used to determine the direction of change, denoted as down for the negative value and up for the positive value. The genes were ranked on *p*-values, logFC and the FDR. We used a volcano plot to visualize the distribution of *p*-values and logFC resulting from comparison of gene expression levels within disease and between the two diseases. Genes were ranked on *p*-values, FDR and logFC.

To determine whether genes associated with both COVID-19 and lung cancer are co-regulated and have similar patterns of expression profiles, we performed hierarchical clustering using the Pearson correlation coefficient as the measure of distance between pairs of genes and complete linkage as the clustering method. Hierarchical clustering was performed using Morpheus [27]. To identify molecular networks and signaling pathways associated with the two diseases, we performed network and pathway analysis using the Ingenuity Pathways Analysis (IPA) software [28]. We mapped the up and downregulated genes that were highly associated with both diseases onto networks and canonical pathways. We used Fisher’s exact t-test in network and pathways analysis to compute estimates of *p*-values. Additionally, we computed the Z-scores to assess the likelihood and reliability of correctly predicting molecular networks to which the genes belonged. The FDR was used to correct for multiple hypothesis testing in pathway analysis [26]. The predicted molecular networks and signaling pathways were ranked based on Z-scores and log *p*-values, respectively. To characterize the molecular functions, biological processes and cellular components in which the genes associated with the two diseases are involved, we performed gene ontology analysis [29], as implemented in IPA [28].

## 3. Results

Clinical management of lung cancer patients in the COVID-19 pandemic era poses significant challenges. One of the more significant challenges has been the lack of information about the molecular mechanisms underlying the association between the two diseases. This knowledge gap has the potential to disrupt essential oncological services provided to lung cancer patients and lead to suboptimal care with potentially deadly consequences. To address this key knowledge gap and critical unmet medical need, we performed integrative genomic data analysis combining gene expression data from lung tissues derived from COVID-19-affected and lung-cancer-affected individuals to discover a signature of genes associated with both diseases, signatures of genes uniquely associated with each disease and molecular networks and signaling pathways shared by the two diseases. Our findings are summarized in the subsections below.

### 3.1. Discovery of Signatures of Genes Associated with COVID-19 and Lung Cancer

To discover a signature of genes transcriptionally associated with COVID-19 and a signature of genes transcriptionally associated lung cancer, we compared gene expression levels between lung samples derived from patients severely affected by COVID-19 and normal lung samples, and between lung tumors and normal lung samples. 

**Figure 2:**
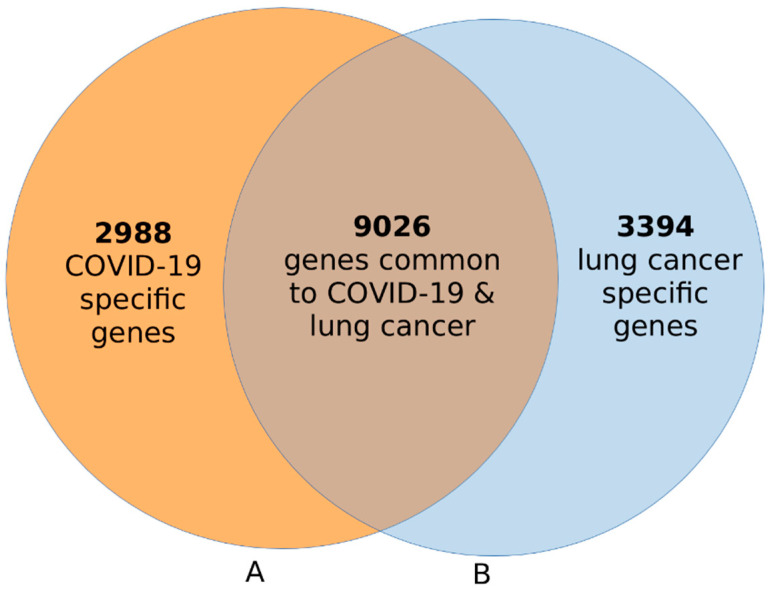
Venn diagram showing signatures of genes uniquely associated with COVID-19 ((**A**), orange) and a signature of genes associated with lung cancer ((**B**), blue). A signature of 9026 genes associated with both diseases is shown in the intersection.

The results of this investigation are summarized in a Venn diagram in Figure 2. A comparison of gene expression levels between COVID-19-affected lung and normal lung tissue samples revealed a signature of 12,014 significantly (*p* < 0.05) differentially expressed genes associated with COVID-19 (Figure 2A). The distribution of estimates of *p*-values and logFC for all the 12,014 genes is presented in a volcano plot in supplementary Figure SF1A. A complete list of all 12,014 significantly (*p* < 0.05) differentially expressed genes associated with COVID-19, along with their estimates of *p*-values and logFC, are presented in supplementary Table S1A. 

The same analysis, comparing gene expression levels between lung cancer and normal lung samples, revealed a signature of 12,420 significantly (*p* < 0.05) differentially expressed genes associated with lung cancer (Figure 2B). The distribution of estimates of *p*-values and logFC for all the 12,420 genes is presented in a volcano plot shown in the supplementary Figure SF1B. A complete list of all the 12,420 genes that were significantly (*p* < 0.05) associated with lung cancer is presented in supplementary Table S1B. Overall, the investigation confirmed our hypothesis that transcription profiling using lung samples from COVID-19- and lung-tumor-affected individuals could identify signatures of genes associated with each disease (Figure 2).

The primary goal of the investigation was to identify a signature of genes associated with both COVID-19 and lung cancer. To achieve this goal, we combined the set of genes associated with COVID-19 with the set of genes associated with lung cancer and sorted them by estimates of *p*-values and logFC. If the signature of genes was significant in both diseases, as measured by the estimated *p*-value (*p* ≤ 0.05), it was considered to be associated with both diseases. Thus, molecular crosstalk perturbation between COVID-19 and lung cancer in this portion of the investigation was measured by discovering a signature of genes associated with or shared by both diseases. 

The results of this investigation are summarized in a Venn diagram in Figure 2. The investigation revealed a signature of 9026 genes transcriptionally associated with both COVID-19 and lung cancer, confirming our hypothesis (Figure 2, see intersection). In addition, the investigation revealed a signature of 2988 genes associated with COVID-19 only (Figure 2A) and a signature of 3394 genes associated with lung cancer only (Figure 2B). Interestingly, a majority of the genes were associated with both diseases.

### 3.2. Changes in Expression Profiles for Genes Associated with COVID-19 and Lung Cancer 

Following the discovery of a signature of 9026 genes associated with both COVID-19 and lung cancer, we conducted additional investigation to determine their differences in patterns of expression and direction of change. We addressed this issue by comparing the expression levels of the 9026 genes between COVID-19 and lung cancer. Note that this analysis framework was crucial in identifying genes with different patterns of expression profiles (i.e., genes upregulated in COVID-19 and downregulated in lung cancer and vice versa). The differences in patterns of expression were determined by the estimates of *p*-values, whereas the direction of change was determined by the logFC, represented by a negative value for downregulation and positive values for upregulation.

Comparison of gene expression profiles produced a signature of 7599 significantly (*p* < 0.05) differentially expressed up and downregulated genes associated with both COVID-19 and lung cancer. The remaining 1427 genes did not show differences in patterns of expression profiles between the two diseases. The distribution of estimates of *p*-values and logFC for all the 7599 genes is presented in a volcano plot in supplementary Figure SF2. Among the significantly differentially expressed up and downregulated genes, 4124 genes were upregulated and 3475 were downregulated between COVID-19 and lung cancer tumors. A list of the top 50 most highly significantly differentially expressed (25 up and 25 downregulated) genes along with their estimates of *p*-values and logFC is presented in Table 1. A complete list of all the 7599 genes showing differences in patterns of expression profiles between COVID-19 and lung cancer along with their estimates of *p*-values, logFC and direction of change (up/down) is presented in supplementary Table S2. Overall, the investigation revealed crosstalk in molecular perturbation between COVID-19 and lung cancer. 

### 3.3. Similarity in Expression Profiles for Genes Associated with Both COVID-19 and Lung Cancer

Quantitative assessment of differences in gene expression levels provides limited information about the regulatory patterns of the genes perturbed in both diseases. Genes associated with both diseases could still behave differently in each disease. Therefore, to characterize the patterns of gene expression profiles and their direction of change among the genes associated with both COVID-19 and lung cancer, we performed hierarchical clustering, as explained in the Materials and Methods section. We hypothesized that, among the genes associated with both COVID-19 and lung cancer, there are differences in their patterns of expression profiles in the two diseases. Here, we sought to discover genes that were upregulated in lung cancer and downregulated in COVID-19, and genes that were upregulated in COVID-19 and downregulated in lung cancer. For this analysis, we used the top 515 most highly significantly (*p* < 10^−48^) differentially expressed up and downregulated genes associated with both diseases. Note that these genes were selected from the 7599 up and downregulated genes significantly associated with both diseases. Thus, this analysis includes the genes in Table 1. The selection of the top 515 genes used for hierarchical clustering was crucial to eliminate any spurious patterns of expression profiles.

**Figure 3:**
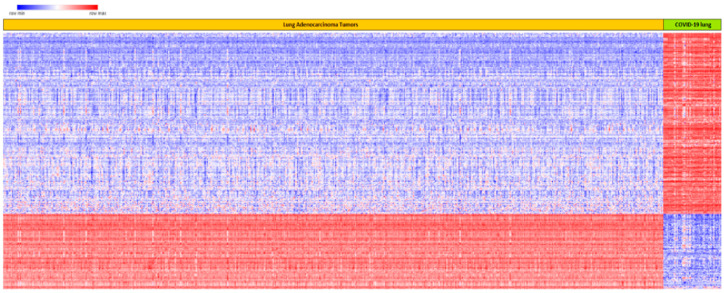
Patterns of expression profiles for the top 515 upregulated and downregulated genes in lung cancer and in COVID-19, generated using hierarchical clustering on genes associated with both diseases. Genes are represented in rows, and lung cancer and COVID-19 samples in columns. Red color indicates upregulation and blue represents downregulation.

The results showing patterns of expression profiles for all the 515 up and downregulated genes associated with both COVID-19 and lung cancer are presented in Figure 3. Owing to space limitations, the names of genes are not presented in Figure 3, as they could not fit in the figure. As shown in Figure 3, hierarchical clustering produced two clusters of genes: a cluster of genes that were upregulated in lung cancer and downregulated in COVID-19, and a cluster of genes that were upregulated in COVID-19 and downregulated in lung cancer (Figure 3). This confirmed our hypothesis that, among the genes associated with both COVID-19 and lung cancer, there are differences in patterns of their expression profiles in the two diseases. Out of the 515 genes evaluated, 363 genes were upregulated in COVID-19 and downregulated in lung cancer (Figure 3). The other 152 were downregulated in COVID-19 and upregulated in lung cancer (Figure 3). A complete list of all the 515 up and downregulated genes is provided in supplementary Table S3. Overall, this portion of the investigation further confirmed the crosstalk in molecular perturbation between COVID-19 and lung cancer by showing that genes associated with both are co-regulated and have similar patterns of expression profiles. 

### 3.4. Discovery of Molecular Networks and Signaling Pathways Shared by the Two Diseases

To gain insights about the broader biological context in which genes associated with both lung cancer and COVID-19 operate and to determine whether they share the same regulatory programs, we performed network analysis. We hypothesized that genes associated with both COVID-19 and lung cancer are functionally related and interact in gene regulatory networks. We sought to identify molecular networks associated with both COVID-19 and lung cancer and to characterize molecular functions, biological and disease processes, and cellular components in which they are involved. This framework was crucial to determining whether these genes share the same regulatory mechanisms. For this investigation, we mapped the top 515 up and downregulated genes that were co-regulated and highly significantly associated with both diseases onto the networks, as described in the Materials and Methods section. 

**Figure 4:**
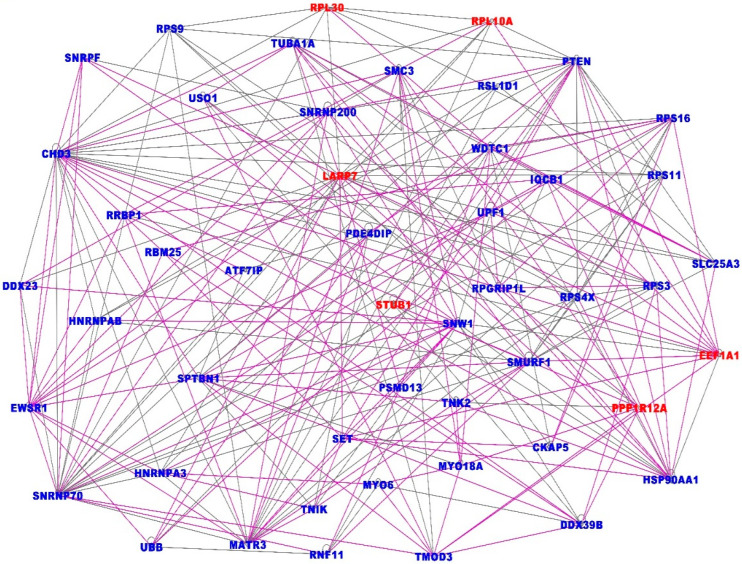
Highly interconnected gene regulatory networks associated with both COVID-19 and lung cancer. The gene names in red font indicate upregulation and blue font downregulation. The solid lines indicate overlapping functions between the genes in the seven merged networks.

The investigation revealed 25 gene regulatory networks with Z-scores ranging from 10 to 55, containing genes with overlapping functions. The results showing the top seven gene regulatory networks (merged) are presented in Figure 4. Note that to ensure easy presentation and clarity of networks, only the most interconnected genes (≥3 connections) in the networks are presented. Genes and networks with fewer interactions were pruned to remove spurious interactions. The top seven networks (Figure 4) with Z-scores 40 to 55 contained genes predicted to be involved in organismal injury and abnormalities, gene expression, protein synthesis, RNA damage and repair, connective tissue disorders, amino acid metabolism, cellular assembly and organization, small molecule biochemistry, cancer, DNA replication, recombination and repair, gastrointestinal disease and protein synthesis. 

In addition, the analysis revealed gene regulatory networks containing genes predicted to be involved in cell-to-cell signaling and interaction, infectious diseases, ophthalmic disease, hematological disease, immunological disease, RNA post-transcriptional modification, cardiac arteriopathy, cardiac fibrosis, cardiovascular disease, cardiac dilation, cell cycle, cellular assembly and organization, respiratory disease, cell-mediated immune response, cellular movement, cellular function and maintenance, lipid metabolism, drug metabolism, molecular transport, organ morphology, tissue development, tissue morphology, cell morphology, cellular function and maintenance, cell death and survival, inflammatory response, organismal survival, carbohydrate metabolism. A complete list of all the predicted molecular networks, the genes they contain and the top diseases and molecular functions they are involved in, is presented in supplementary Table S4. 

Overall, the results showed that COVID-19 and lung cancer share the same regulatory mechanisms and that network analysis is a powerful approach for revealing molecular crosstalk perturbation between lung cancer and COVID-19. Taken together, the investigation demonstrated that the association between COVID-19 and lung cancer can be considered an emergency property of molecular networks encompassing many functionally related genes, as opposed to the core biological processes driving the association between the two diseases being driven by responses to molecular perturbation in a small number of genes. 

To determine whether COVID-19 and lung cancer share the same regulatory mechanisms and signaling pathways, gain further insights about the broader biological context in which genes associated with both diseases operate and discover potential therapeutic targets, we mapped the top 515 up and downregulated genes that were highly significantly associated with both diseases onto canonical pathways. 

**Figure 5:**
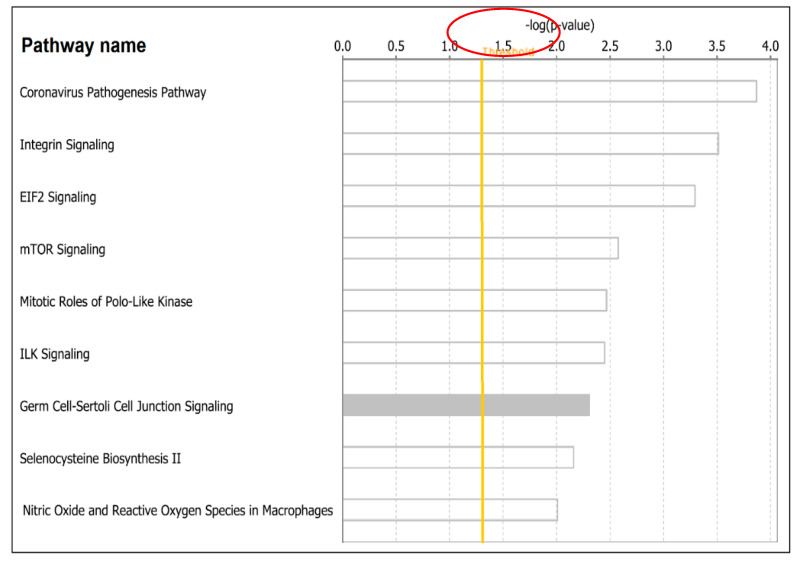
Signaling pathways associated with both COVID-19 and lung cancer represented as bars. The orange solid line indicates the threshold above which a signaling pathway was declared significantly associated with both diseases, as determined by the –log(*p*-values) shown on the x-axis above. The y-axis shows names of signaling pathways associated with both diseases.

The results showing the top nine most highly significant signaling pathways associated with both COVID-19 and lung cancer are presented in Figure 5. The top pathways discovered included: the Coronavirus Pathogenesis signaling pathway -log(*p*-value, 3.87E), which contained the genes *DDIT3*, *E2F6*, *EEF1A1*, *RPS11*, *RPS16*, *RPS3*, *RPS4X*, *RPS9*, *SERPINE1*, *STING1*, *SUV39H1*, *TGFBR2*; the Integrin signaling pathways -log(*p*-value, 3.51E), containing genes *FNBP1*, *PAK2*, *PARVA*, *PARVB*, *PIK3R2*, *PLCG2*, *PPP1R12A*, *PTEN*, *RHOD*, *RND3*, *TLN2*, *TNK2*, *TSPAN3*, *TTN*; the EIF2 signaling pathways -log(*p*-value, 3.29E), containing genes *DDIT3*, *EIF1*, *PIK3R2*, *PPP1CA*, *RPL10A*, *RPL22L1*, *RPL30*, *RPL32*, *RPL36*, *RPS11*, *RPS16*, *RPS3*, *RPS4X*, *RPS9*; the mTOR signaling -log(*p*-value, 2.58E), containing genes *FNBP1*, *PIK3R2*, *RHOD*, *RICTOR*, *RND3*, *RPS11*, *RPS16*, *RPS3*, *RPS4X*, *RPS6KA1*, *RPS9*, *ULK1*. 

Additional signaling pathways associated with both diseases discovered included: the Mitotic Roles of Polo-Like Kinase -log(*p*-value, 2.47E), containing genes *CDK1*, *HSP90AA1*, *PRC1*, *SLK*, *SMC3*, *STAG2*; the ILK signaling -log(*p*-value, 2.45E), containing genes *FNBP1*, *PARVA*, *PARVB*, *PIK3R2*, *PPP1R12A*, *PTEN*, *RHOD*, *RICTOR*, *RND3*, *TMSB10/TMSB4X*, *VIM*; the Germ Cell-Sertoli Cell Junction signaling -log(*p*-value, 2.30E), containing genes *FNBP1*, *JUP*, *MAP3K3*, *PAK2*, *PIK3R2*, *RHOD*, *RND3*, *TGFBR2*, *TJP1*, *TUBA1A*; the Selenocysteine Biosynthesis II -log(*p*-value, 2.16E), containing genes *SARS2*, *SEPHS1*; and Production of Nitric Oxide and Reactive Oxygen Species in Macrophages -log(*p*-value, 2.01E) containing genes *FNBP1*, *MAP3K3*, *MPO*, *PIK3R2*, *PLCG2*, *PPP1CA*, *PPP1R12A*, *RHOD*, *RND3*, *SERPINA1*. A complete list of all predicted pathways containing genes associated with both diseases is presented in supplementary Table S5. 

In summary, an integrative analysis combining gene expression data from COVID and lung cancer samples produced a signature of genes, molecular networks and signaling pathways associated with both diseases, confirming the crosstalk in molecular perturbation between the two diseases. Thus, in the context of common human diseases, molecular crosstalk perturbation between COVID-19 and lung cancer can be considered an emergent property of molecular networks and signaling pathways associated with both diseases, as opposed to the core biological processes associating the two diseases being driven by responses to changes in a small number of genes dysregulated in only one disease. Taken together, the investigation demonstrates that integrating large-scale, high-dimensional transcriptomic data holds promise to discover potential drivers of the severity of COVID-19 in individuals with lung cancer and targets for the development of therapeutic targets.

## 4. Discussion

Patients with lung cancer have the worst outcomes when affected by COVID-19 [6,7]. The molecular mechanisms associating COVID-19 with lung cancer are not known. This investigation was conducted to address this knowledge gap. The investigation revealed a signature of genes, molecular networks and signaling pathways associated with both diseases. These findings suggest that COVID-19 and lung cancer have shared regulatory mechanisms. This integrative genomics data analysis framework provides the first step and is crucial to discovering the molecular drivers of COVID-19 severity in individuals with lung cancer and the discovery of potential therapeutic targets. To our knowledge, this is the first study to map the landscape of molecular crosstalk perturbation between COVID-19 and lung cancer. 

A number of epidemiological studies revealing poorer outcomes for COVID-19 in lung cancer patients have been reported [30,31]. Those poorer outcomes have been attributed to the compromised immune system resulting from chemotherapy treatment, which lung cancer patients undergo, a risk factor to SARS-CoV-2 infection [30,31]. The novel aspect of our investigation is that it provides new knowledge by discovering a signature of genes, molecular networks and signaling pathways associated with both diseases. This has not been previously reported. To the extent that imbalance in host immune response to SARS-CoV-2 drives the development and progression of COVID-19 [32,33], the genes and pathways discovered in this investigation, if confirmed, could serve as potential clinically actionable molecular markers and therapeutic targets. For example, the immune-responsive cytokines and pro-inflammatory genes and the signaling pathways they control associated with both diseases discovered in this investigation could serve as molecular markers to guide clinical management of individuals with lung cancer affected by COVID-19 [7,34] and the development of novel, more effective therapeutics [35,36,37,38,39]. 

Some of the major challenges in clinical management of COVID-19 include extrapulmonary manifestations of the disease and its effects on multiple organs, including the lungs [40,41,42]. Extrapulmonary manifestations include thrombotic complications, myocardial dysfunction and arrhythmia, acute coronary disease syndromes, acute kidney injury, gastrointestinal symptoms, hepatocellular injury, hyperglycemia and ketosis, neurologic illnesses, ocular symptoms and dermatologic complications [40,41,42,43]. Although we did not investigate the association of the discovered genes with extrapulmonary manifestations in COVID-19, the discovery of genes with multiple overlapping functions involved in many biological processes suggests that some of the identified genes and gene regulatory networks may be involved in extrapulmonary activities. Moreover, the lung as an organ is likely to function in unison with other organs. Under such conditions, the effects of COVID-19 on the lungs have potential to trigger a cascade of events likely to affect other organs and lead to extrapulmonary manifestations. Indeed, lungs as organs contain many cells that can play many different roles. Although we did not examine individual lung cells, previous studies have shown that transcription profiling could reveal novel mechanisms of SARS-CoV-2 infection in human lung cells [44,45]. 

Another finding of significance in this investigation was the discovery of gene regulatory networks and signaling pathways associated with both diseases. This suggests that the host–pathogen interactions linking the two diseases are complex. The novel aspect and clinical significance of this finding is that it could increase our understanding of host–pathogen interactions, a critical step in vaccine and drug development [46]. For example, the discovery of the coronavirus pathogenesis signaling pathway in this study has the promise to increase our understanding of the pathogenesis of COVID-19 and the molecular mechanisms driving the disease. Although signatures of genes associated with COVID-19 have been reported [17,21], molecular crosstalk perturbation between COVID-19 and lung cancer has not been reported. This framework is crucial for understanding the SARS-CoV-2 host interactions and discovering the molecular mechanisms driving disease severity and poorer outcomes in individuals with lung cancer impacted by COVID-19. Success in understanding the link between COVID-19 and lung cancer has the promise to ensure no disruption to essential oncological services and could guarantee optimal care in lung cancer patients in the COVID-19 pandemic era. 

The discovery of the integrin signaling pathway associated with both lung cancer and COVID-19 has clinical significance. In cancer, integrins mediate cell adhesion and transmit mechanical and chemical signals to the cell interior [47]. Deregulation of integrin signaling in cancer empowers tumor cells with the ability to proliferate without restraint and to survive in foreign microenvironments [47]. Integrin signaling drives multiple stem cell functions, including tumor initiation, epithelial plasticity, metastatic reactivation and resistance to oncogene- and immune-targeted therapies [47]. These mechanisms of integrin regulation have the potential to provide a gateway for COVID-19 to drive its adverse effects on lung cancer patients. Thus, the integrin signaling pathway could serve as a potential therapeutic target. The discovery of the mTOR signaling pathway was of particular interest because the application of PI3K-Akt-mTOR signaling axis to COVID-19 disease and to other chronic conditions, such as obesity, has been reported [48,49]. This is a significant finding because patients with lung cancer, obesity and related chronic diseases affected by COVID-19 tend to have poorer outcomes [49,50], which suggests that this pathway has the promise to serve as a therapeutic target. 

The discovery of some rather unexpected connections, such as ophthalmic disease and cardiac fibrosis, in network analysis was of particular interest. Cardiac involvement in patients who recovered from COVID-19 has been reported [51]. Recently, cardiopulmonary recovery after COVID-19 has been reported in a prospective multicenter trial [52]. Ophthalmic manifestations of COVID-19 have been reported [53]. Although the molecular mechanisms associating ophthalmic disease and cardiac fibrosis with COVID-19 are not well characterized, the connections observed in this investigation could partially be explained by the functional versatility of identified key genes.

This investigation shows that COVID-19 and lung cancer have shared regulatory programs and signaling pathways. However, the limitations of the study must be acknowledged. The investigation used data from COVID-19- and lung-cancer-affected individuals, not patients affected by both diseases. In addition, we did not perform mechanistic experiments to confirm the results from computational analysis. This was because such data were not available. With these limitations in mind, the manuscript emphasizes modeling the biological association between COVID-19 and lung cancer and considers this the first step in a long road to discovery of the molecular mechanisms driving the two diseases and adverse outcomes. Such line of research would require molecular and clinical data on individuals affected by both diseases. In addition, experimental confirmation of genomic discoveries would be necessary. That framework will be crucial to ensure the translation of genomic discoveries into clinical practice to improve clinical management of lung cancer patients in the COVID-19 pandemic era. 

## 5. Conclusions

A key knowledge gap and critical unmet medical need in the clinical management of lung cancer patients in the COVID-19 pandemic era is the characterization of molecular mechanisms associating the two diseases. Using an integrative genomic data analysis approach, combining gene expression data from individuals affected by COVID-19 and individuals affected by lung cancer, we discovered a signature of genes, molecular networks and signaling pathways associated with both diseases. The investigation demonstrated that integrative data analysis, combining transcriptomic data from COVID-19 and lung cancer, is a powerful approach to deciphering the molecular mechanisms linking the two diseases. Further research on a population affected by both COVID-19 and lung cancer and experimental confirmation of the results is recommended to discover molecular drivers of the association between the two diseases, clinically actionable biomarkers and potential therapeutic targets. Such an investigation will be crucial to ensuring the translation of genomic discoveries in clinical practice to improve essential oncological services and guarantee the optimal care of lung cancer patients in the COVID-19 pandemic era. 

## 6. Patents

No patents resulted from the work reported in this manuscript.

## Figures and Tables

**Table 1:** Top 25 upregulated and top 25 downregulated differentially expressed genes between COVID-19 and lung cancer tumors and their estimates of *p*-values and logFC.

Gene Name	Chromosome	logFC	*p*-Value	Up/Downregulated
SNRNP200	2q11.2	−7.66807	1.00 × 10^−300^	Down
MT-CO1	9q21.31	−7.45498	1.00 × 10^−300^	Down
MT-CO2	20q13.33	−6.52755	1.00 × 10^−300^	Down
SHOC2	10q25.2	−7.37305	1.69 × 10^−300^	Down
KRT19	17q21.2	−8.23486	6.20 × 10^−290^	Down
RPS3	11q13.4	−9.84811	2.78 × 10^−289^	Down
ZSWIM6	5q12.1	−6.9536	3.27 × 10^−275^	Down
SFPQ	1p34.3	−10.5243	4.12 × 10^−274^	Down
SERPINA1	14q32.13	−8.07982	1.13 × 10^−273^	Down
PAK2	3q29	−5.11624	9.35 × 10^−267^	Down
VPS35	16q11.2	−5.93836	1.86 × 10^−264^	Down
ANKLE2	12q24.33	−7.79146	1.73 × 10^−260^	Down
WDR1	4p16.1	−5.53354	5.42 × 10^−260^	Down
SORL1	11q24.1	−7.31638	2.00 × 10^−258^	Down
ZC3H11A	1q32.1	−4.42734	4.58 × 10^−257^	Down
ZCCHC2	18q21.33	−7.37741	5.73 × 10^−255^	Down
GOLGB1	3q13.33	−10.4188	1.95 × 10^−251^	Down
ZFHX3	16q22.2	−7.58576	1.35 × 10^−247^	Down
MIER1	1p31.3	−7.31554	1.16 × 10^−246^	Down
P4HB	17q25.3	−6.7194	1.90 × 10^−246^	Down
TBC1D2B	15q24.3	−8.07794	3.29 × 10^−246^	Down
SIPA1L1	14q24.1	−7.41523	6.22 × 10^−244^	Down
CRTAP	3p22.3	−7.55263	5.75 × 10^−240^	Down
LITAF	16p13.13	−6.36823	1.43 × 10^−239^	Down
TANC1	2q24.2	−4.77239	5.00 × 10^−234^	Down
PCDHB13	5q31.3	6.96034	1.94 × 10^−161^	Up
SYNDIG1	20p11.21	6.746343	1.70 × 10^−160^	Up
LINC00324	17p13.1	6.797806	1.38 × 10^−157^	Up
LEKR1	3q25.31	9.303897	1.01 × 10^−150^	Up
TMEM59L	19p12	7.908565	2.29 × 10^−143^	Up
SERP2	13q14.11	7.323597	1.24 × 10^−141^	Up
RTN4IP1	6q21	6.36925	6.08 × 10^−139^	Up
ZDHHC19	3q29	7.455968	1.05 × 10^−136^	Up
SEC61A2	10p14	5.899905	1.34 × 10^−133^	Up
TPT1-AS1	13q14.13	7.189802	3.80 × 10^−133^	Up
EGFL6	Xp22.2	7.885459	2.92 × 10^−128^	Up
SPDEF	6p21.31	6.341318	2.09 × 10^−124^	Up
SARS2	19q13.2	6.014209	3.10 × 10^−122^	Up
MUC13	3q21.2	7.0324	3.30 × 10^−120^	Up
SNX32	11q13.1	5.567126	8.27 × 10^−119^	Up
RPS13P2	1p32.3	7.698321	4.21 × 10^−118^	Up
ZNF208	19p12	4.720081	2.03 × 10^−117^	Up
UBASH3A	21q22.3	6.09295	1.18 × 10^−114^	Up
TMEM128	4p16.3	5.123725	4.21 × 10^−110^	Up
SEC31B	10q24.31	4.168494	4.12 × 10^−106^	Up
PRC1	15q26.1	7.932253	2.82 × 10^−104^	Up
SNRNP25	16p13.3	5.221731	2.26 × 10^−103^	Up
PCSK9	1p32.3	4.906412	5.31 × 10^−103^	Up
TCF15	20p13	5.559709	1.73 × 10^−100^	Up
GREB1	2p25.1	6.243165	1.08 × 10^−99^	Up

## Data Availability

Original RNA-Seq/gene expression data and clinical information on COVID-19 are available at the Gene Expression Omnibus (GEO) https://www.ncbi.nlm.nih.gov/geo/; database under accession # GSE150316. Original RNA-Seq/gene expression data and clinical information on lung cancer and controls used in this study were downloaded from The Cancer Genome Atlas (TCGA) via the Genomics Data Commons and are available at: https://www.cancer.gov/about-nci/organization/ccg/research/structural-genomics/tcga and are accessible via the Genomics Data Commons GDC https://gdc.cancer.gov/; Additional data are shared through supplementary tables referenced in the manuscript and listed below and provided as supplementary material to this report.

## Supplementary Materials

The following supporting information can be downloaded at: https://www.mdpi.com/article/10.3390/ijerph19063454/s1, Supplementary Figure SF1A. Volcano plot showing differentially expressed genes between COVID-19-affected lungs and normal lung tissue samples. Supplementary Figure SF1B. Volcano plot showing differentially expressed genes between lung adenocarcinoma tumors and normal lung tissue samples. Supplementary Figure SF2. Volcano plot showing differentially expressed genes between lung cancer and COVID-19. Supplementary Table S1A. List of the 12,014 significantly differentially expressed genes associated with COVID-19-affected lung tissue. Supplementary Table S1B. List of the 12,420 significantly differentially expressed genes associated with lung cancer. Supplementary Table S2. List of the 7599 significantly differentially expressed genes associated with both COVID-19 and lung cancer. Supplementary Table S3. List of the top 517 most highly significantly differentially expressed genes associated with both COVID-19 and lung cancer. These genes were used in heat map and pathway analysis. Supplementary Table S4. Predicted molecular networks and genes associated with both COVID-19 and lung cancer along with information on top diseases and molecular functions in which they are involved. Supplementary Table S5. Predicted signaling pathways and genes associated with both COVID-19 and lung cancer.
