# Supplementary material for: Landscape of Molecular Crosstalk Perturbation between Lung Cancer and COVID-19
Source: Int J Environ Res Public Health. 2022 Mar 15;19(6):3454. doi: 10.3390/ijerph19063454 (PMC8953719; doi:10.3390/ijerph19063454)
Supplement: Supplementary file 1 [file ijerph-19-03454-s001.zip › ADITI-HICKS-FV-Supplementary Figures-6FEB2022.pdf]

Supplementary Figures

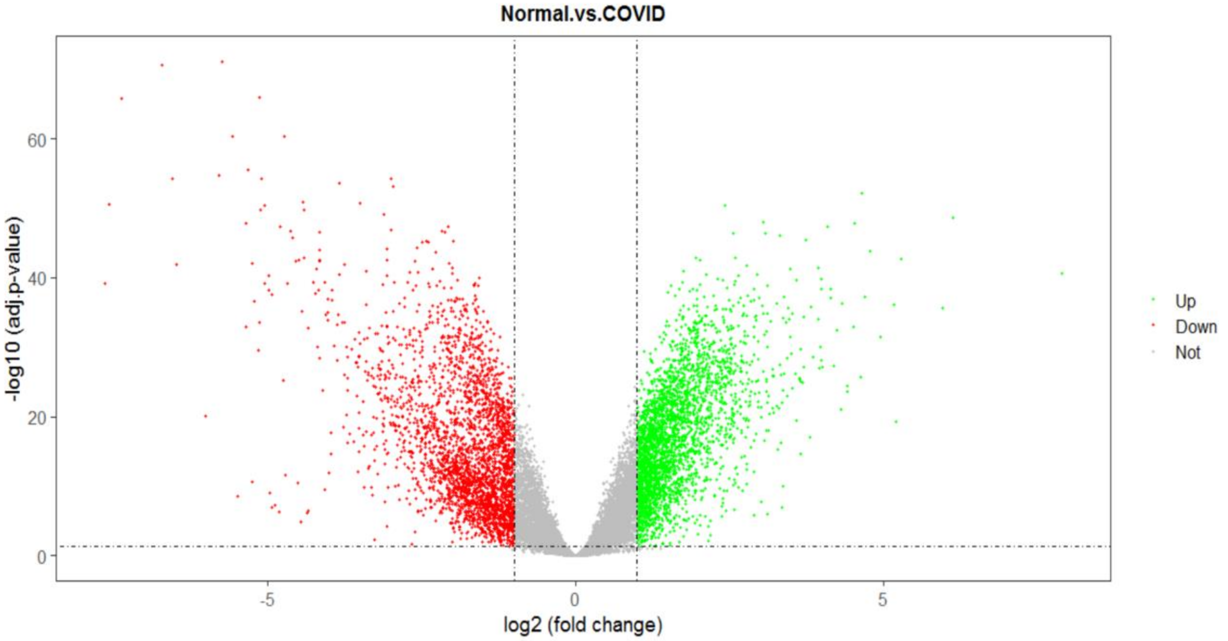

Supplementary Figure SF1 (A). Volcano plot showing differentially expressed genes between COVID-19 affected lungs and normal lung tissue samples. Red color indicates down regulated and green upregulated significantly differetially expressed. Gray color indicates not differentially expressed.

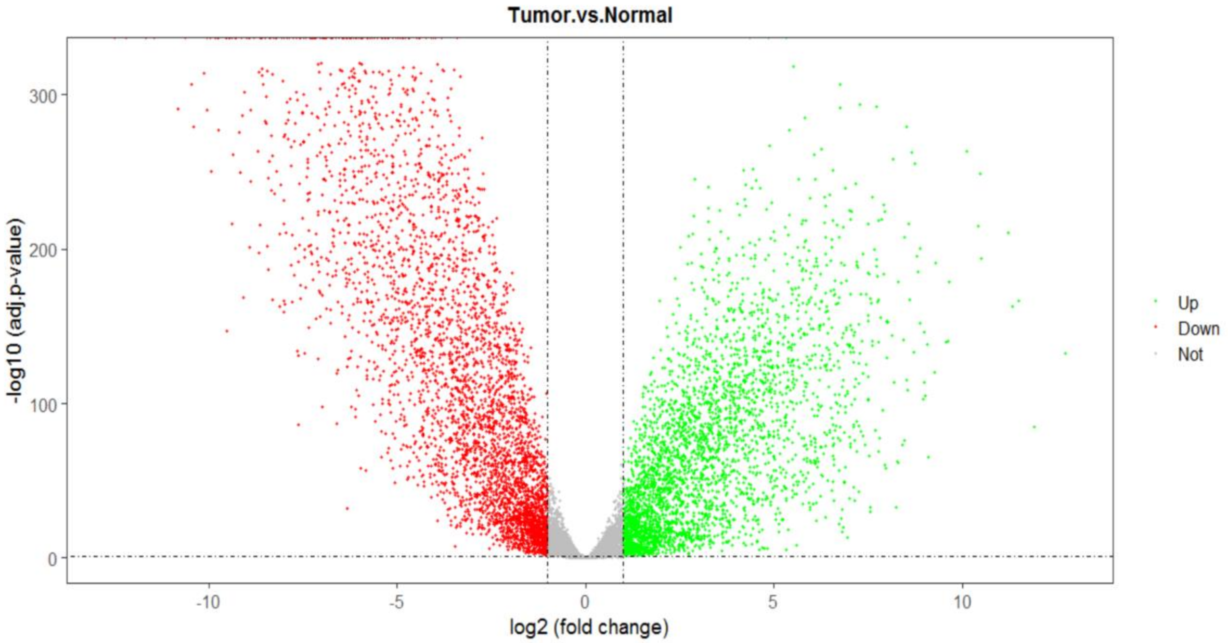

Supplementary Figure SF1 (B). Volcano plot showing differentially expressed genes between lung adenocarcinoma tumors and normal lung tissue samples. Red color indicates down regulated and green upregulated significantly differetially expressed. Gray color indicates not differentially expressed.

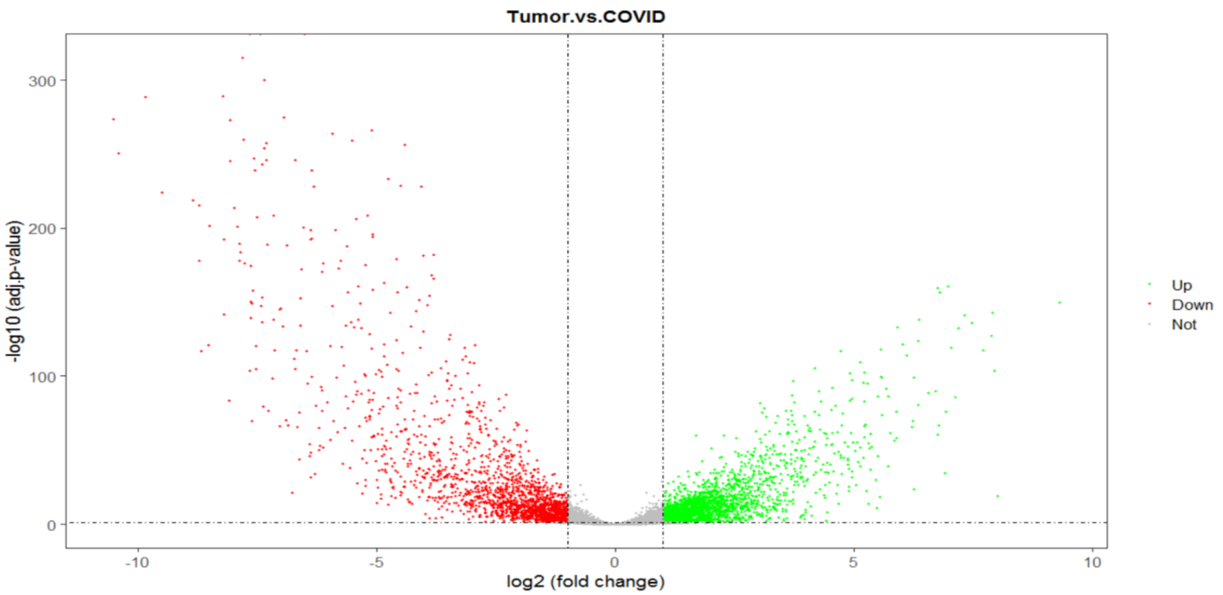

Supplementary Figure SF2. Volcano plot showing differentially expressed genes between lung cancer and COVID-19. Red color indicates down regulated and green upregulated significantly differentially expressed. Gray color indicates not differentially expressed.
